# Influence of Advancing Biological Maturation on Aerobic and Anaerobic Power and on Sport Performance of Junior Rowers: A Longitudinal Study

**DOI:** 10.3389/fphys.2022.892966

**Published:** 2022-05-17

**Authors:** Paulo Francisco de Almeida-Neto, Luiz Felipe Da Silva, Bianca Miarka, Jason Azevedo De Medeiros, Rafaela Catherine da Silva Cunha de Medeiros, Rafael Pereira Azevedo Teixeira, Felipe J. Aidar, Breno Guilherme De Araujo Tinoco Cabral, Paulo Moreira Silva Dantas

**Affiliations:** ^1^ Health Sciences Center, Federal University of Rio Grande do Norte, Rio Grande do Norte, Brazil; ^2^ Physical Education, Natal, Brazil; ^3^ Federal University of Rio Grande do Norte, Natal, Brazil; ^4^ Federal University of Rio de Janeiro, Rio de Janeiro, Brazil; ^5^ Physical Education, Mossoro, Brazil; ^6^ Health Sciences Center, Federal University of Rio Grande do Norte, Rio Grande do Norte, Brazil; ^7^ Federal University of Sergipe, São Cristóvão, Brazil

**Keywords:** physiology, puberty, rowing, athlete performance, sport

## Abstract

**Background:** Researches are linking Biological Maturation (BM) with the performance of adolescent rowers from both genders. Despite this, there is still not enough information about the influence of BM advancement correlating to sports, aerobic and anaerobic performance in adolescent athletes at the sport modality rowing.

**Objective:** Investigate the influence of Biological Maturation on sports performance and aerobic and anaerobic power in adolescent rowing athletes.

**Methods:** A longitudinal observational study, developed over 3 years, with a sample of 52 adolescents, rowing athletes, of both genders (61% male and 39% female) mean age of 16.0 ± 0.5 years old at the start and 18.4 ± 0.5 years old at the end of the study. Analysis was performed once a year. BM was evaluated through maturational groups generated from Age Peak Height Velocity; maximum aerobic power [VO_2_Max (ml/kg/min)] and mean anaerobic power (Watts) through the ergometer test (indoor rowing); peak anaerobic power (Watts) through a mathematical model derived from competition time, to determine sports performance analyzed the race time during world championship tryouts.

**Results:** The advancement of BM influenced the reduction of the test time and increase of the mean anaerobic power (Watts) in indoor rowing (η2*p* > 0.36, *p* < 0.05), as well as an improvement in performance in sports competition (η2*p* > 0.35, *p* < 0.05). However, the advancement of BM did not affect VO2Max (ml/kg/min) in young elite rowing athletes of both sexes (*p* > 0.05).

**Conclusion:** Advances in biological maturation have been shown to influence the anaerobic and sports performance (reduction of the execution time in 2,000-m) of adolescent rowing athletes of both genders.

## Graphical Abstract

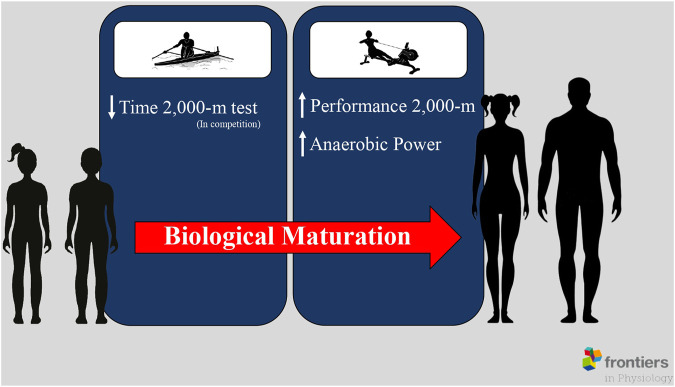

## 1 Introduction

Rowing is an Olympic sport whose rowers have recorded high oxygen consumption rates (72 ml kg-1 min-1 in men) and average peak power of 629 ± 51 W in elite male athletes (Nevill et al., 2003; Egan-Shuttler et al., 2019). The distances covered in a race can range from 1,000-m, 1,500-m, 2,000-m, to 6,000-m (Mikulic, 2009; Gee et al., 2012; Akça, 2014; Maciejewski et al., 2016). Moreover, to promote vessel displacement, muscle contraction from the human body requires the use of energy demands from oxidative pathways (i.e., which use oxygen) and glycolytic pathways (i.e., which use glycogen) (McNarry and Barker, 2018).

A 2,000-m rowing race has three phases: start (first 500-m), central (after 500-m to 1,700-m), and finish (last 300-m) (Nor, 2000). During the start, athletes use high stroke rates to increase the vessel’s displacement speed. Thus, to achieve and maintain speed, energy from the glycolytic pathway is predominantly used (Gomes et al., 2015; Martin and Tomescu, 2017; Skorski and Abbiss, 2017). And this is because muscle cells are operating for a short time to acquire enough oxygen for energy production (Billat et al., 2009).

The predominance of glycolytic metabolism can be explained by the hypothesis of motor unit recruitment (Moritani and Takaishi et al, 1993), in which the imposed intensity requires greater recruitment of type II (glycolytic) motor units. They predominantly have the isoenzyme of Lactate Dehydrogenase (LDH), which favors the conversion of pyruvate to lactate, and a more significant presence of the mATPase isophome hydrolyzes more ATP than type I fibers (Han et al., 2003). For this reason, rapid ATP resynthesis is required, which is not characteristic of the oxidative pathway (Azevedo et al., 2009).

During the starting phase, there is a natural reduction in the anaerobic potency of the athletes (attributed to the accumulation of hydrogen ions and a drop in the intramuscular pH that culminates in the decrease in the activity of critical glycolytic enzymes, such as phosphofructokinase (PFK) (Hargreaves and Spriet, 2006) forcing them to enter the major phase (i.e., the period between the start and completion of the race). There is characterized by a reduction in stroke rates, parallel to the metabolic level, where the glycolytic energy system reduces its contribution and energy production becomes greater predominantly by aerobic pathways (between 65%–75%) (Martin and Tomescu, 2017). A possible muscular adaptation in mitochondrial activity may provide rapid recovery of the phosphagen system through rapid phosphocreatine resynthesis (Hargreaves and Spriet, 2020). This contributes to an increase in stroke rates in the finishing phase of the race so that the anaerobic metabolism returns to the predominance of energy production (Held et al., 2020).

Understanding the phases of a rowing event and the importance of the variation between anaerobic and aerobic metabolism is essential to address factors influencing cellular energy production. It is known that, regardless of age and gender, total body mass and level of physical fitness can promote changes in the efficiency of cellular energy production in the human body (Rowland, 2008; McNarry and Barker, 2018). However, when considering adolescent athletes, it is necessary to acknowledge that at this stage of life, the peak of the phenomenon called biological maturation (BM) occurs, which promotes the improvement and maturity of biological systems from birth (for example, musculoskeletal and metabolic) (Scheffler and Hermanussen, 2018).

It is important to note that BM is not always in sync with chronological age and may be delayed or advanced (Moore et al., 2015). Metabolically, the later the BM stage, the greater the efficiency of the oxidative pathway for cellular energy production during physical exercise (Ratel and Blazevich, 2017); as well as, the more advanced the BM stage, the greater efficiency of the glycolytic pathway for production of cellular energy during physical exercise (Rowland, 2008). In agreement, as for the muscle tissue of children and adolescents, when in the delayed BM stage, there is a higher percentage of type I muscle fibers, which exert locomotor functions through the production of oxidative energy (Kriketos et al., 1997; Ratel et al., 2008; Tonson et al., 2010). In this sense, the organism at the delayed BM stage shows higher levels of mitochondrial density and more excellent oxidative enzyme activity (Bell et al., 1980; Ratel and Blazevich, 2017).

In this context, it is suggested that due to children and adolescents in late BM stage often present smaller body structure (such as having a trunk and shorter limbs), favors a faster blood circulation, precisely by reducing the distance between the cardiorespiratory and musculoskeletal system, by promoting gas exchange more rapidly between muscles and lungs (Ratel and Blazevich, 2017). But over the years, mitochondrial density tends to reduce in children and adolescents; that when they reach the advanced maturational stage point to a higher efficiency of anaerobic metabolism (Rowland, 2008).

Previously, lower activity of LDH, Creatine kinase (CK) and Adenylate kinase (AK) enzymes was observed in children compared to adults (Kaczor et al., 2005). Suggesting that advancing chronological age may be responsible for increased activity of anaerobic enzymes. In this regard, it can be conjectured that subjects with advanced maturation indicate higher anaerobic enzyme activity compared to those with late maturation. Similarly, aerobic enzyme activity is higher in late maturing subjects compared to advanced maturing subjects.

Although it is known the characteristics of the metabolism of adolescents in different maturational stages and the characteristics of metabolic demands during the Olympic rowing event, there is still insufficient information on the influence of BM advancement in relation to the aerobic and anaerobic performance of adolescent athletes in rowing sport (Mikulic, 2011). Thus, the aim of this study was to analyze the influence of biological maturation on sports performance and aerobic and anaerobic power in adolescent rowing athletes.

Based on the information in this introduction, our hypothesis was that the advancement of biological maturation would not influence aerobic performance, but would influence the sports and anaerobic performance of adolescent rowing athletes.

## 2 Materials and Methods

### 2.1 Study Design

The study has a longitudinal observational design, where 52 Brazilian rowing adolescent athletes (61% male and 39% female) with a mean age of 16.0 ± 0.5 years old at the beginning of the study and 18.4 ± 0.5 years old at the end of the study, were monitored during 3 years with the completion of three data collections (one per year) prior to the selective for the world rowing championship (in 2019, 2020, and 2021). To determine the sample, athletes ranked among the top 20 in their respective categories (U-17, U-18, and U-19) were searched every year.

To be included, the volunteer must: i) Be affiliated with the confederation and the national rowing federation; ii) At the beginning of the study have a minimum training routine of 2 years; iii) During all 3 years of the research, present a minimum training frequency of five times a week (90 min/session); iv) Not to use any substance that could exert any ergogenic effect on sports performance or biological development (i.e., food supplements such as creatine, caffeine, and taurine; use of illegal drugs to improve performance such as steroids or medical treatment with growth hormone).

Those who: i) had some musculoskeletal limitation to perform the physical tests were excluded; ii) Did not participate in the annual tryouts for the world championships; and iii) Did not participate in the entire follow-up period of the study. iv) Had osteoarticular injuries (i.e., injuries to bone tissue and joints) or muscle injuries that took more than 15 days away from the study period. It is important to emphasize that the weekly and daily training volume did not vary during the study period. The researchers of the present study ensured no modification during the analyses. It was also determined that the study participants did not perform any additional sport during the study period.

In addition, there was a sample loss in males. During the last year of the research, seven athletes did not participate in competitions in individual boats, choosing to participate in double boat tournaments, with the male sample reduced from 31 to 24 subjects.

This study was previously approved by the Ethics and Research Committee of the Federal University of Rio Grande do Norte—Brazil (n: 3,552,010). The present study followed all the protocols of Resolution 466/12 of the National Health Council on 12/12/2012, strictly respecting the Declaration of Helsinki’s national and international ethical principles (Johannes et al., 2017). Furthermore, the present study complied with all the requirements and international standards of the STROBE checklist for observational studies (Von Elm et al., 2014).

### 2.2 Procedures

Initially, participants and their respective guardians were informed about the risks and benefits of participating in the research. Afterward, after the participants and their respective guardians signed the terms of free and informed consent, the monitoring of the athletes was started.

The first round of evaluations was carried out in February 2019, the second in February 2020 (before the COVID-19 pandemic), and due to the setbacks generated by the COVID-19 pandemic, the third round of tests took place in June 2021. It is noteworthy that during the COVID-19 pandemic, the athletes continued training (taking preventive measures and following the guidelines of the national government).

Morphological data (i.e., height, span, and body weight) were collected during the evaluations, and specific performance tests of 2,000-m in indoor rowing and cardiorespiratory power [VO_2_Max (ml/kg/min)] were performed with a digital indoor rowing ergometer. Afterward, the athletes’ sports performance was measured from the competition time (between the fourth and fifth weeks after the performance as mentioned above evaluations). The competitions took place in March 2019 and 2020 and in July in the year 2021. In 2021, the athletes were required to test serology for COVID-19 (all athletes tested negative).

### 2.3 Anthropometry Analysis

The anthropometric evaluations were performed with barefoot subjects wearing only light clothing, where their body mass was measured using a Filizola® (São Paulo, Brazil) digital scale (with a capacity up to 150 kg and a variation of 0.10 kg); for stature, the Sanny® stadiometer (São Paulo, Brazil) (0.1 mm accuracy) was used; the wingspan was measured using a Sanny® anthropometric tape (São Paulo, Brazil). All evaluations were based on the International Society for the Advancement of Kinanthropometry (ISAK) protocol (Silva and Vieira, 2020).

### 2.4 Chronological Age Analysis

The chronological age in months was determined by the sum of the individual’s months of life, from his date of birth to the date of analysis of the present study. In this way, the sum of months of life was divided by 12, resulting in their chronological age in years (Malina and Bouchard, 2002).

### 2.5 Biological Maturation Analysis

Biological maturation was analyzed using the maturational group (MG) calculated from the Age for the Peak Height Velocity (APHV) to find the APHV, we initially determined the Maturity offset through the mathematical models proposed by Moore et al. (2015) (for subjects up to 19 years old). Thus, based on results, we qualitatively classify the stages of biological maturation as follows (Moore et al., 2015): Stage 1) Pre-PHV; Stage 2) Circum-PHV; Stage 3); Post-PHV.

### 2.6 Analysis Performance

After performing a specific 10-min warm-up consisting of a movement circuit performed with bodyweight, the specific performance of the rowers was analyzed using a 2,000-m time trial in an indoor rowing ergometer (Concept® model-D equipped with PM5 digital monitor, FL, United States). The test was carried out in a climate-controlled environment (26°C). The equipment was calibrated following the specifications of the Australian International Rowing Federation concerning predetermined resistance factors based on gender and age group [i.e., U17–U19: male = 125 (Ns^2^/m^2^), female = 100 (Ns^2^/m^2^)] (AUS, 2017). We emphasize that we did not change the resistance factor in the analysis period (from 2019 to 2021). In addition, fans and air conditioners were kept away from the ergometers to avoid air flow blowing directly into the ergometer resistance fans, as this could compromise performance. In the end, the results of the average power in watts and the test time in seconds were assimilated from the equipment by a computer coupled to its PM5 digital monitor. It is important to emphasize that athletes did not have access to their results during the research.

In addition, the result of sports performance in national-level competition (selective for the world championship) was also used as a parameter to analyze performance during the study. During competitions from 2019 to 2021, athletes used Single Scull type boats (model F15, Filippi ® brand, Donoratico, Italy; the weight of 14 kg with a maximum weight-bearing capacity of 75–85 kg; 800 cm long and 29.5 cm wide). We emphasize that for the U-17 category, the distance in the competition event was 1,500-m, and for the U-18 and U-19 categories, the distance performed in the competition event was 2,000-m. For this reason, the test time was used to determine sports performance. In addition to sports performance (in an aquatic environment), the mean power in watts was determined using the Concept 2 ® open-source platform (Concept, 2021), and the peak power in watts was calculated in both sexes by the mathematical model proposed by Almeida-Neto et al. (2020), as shown below:

Peak Power (watts) = {[Body weight (Kg) + Equipment weight (Kg)] × Velocity (m/s)}—22(Kg): kilograms. (m/s): Meters per second.

In both sexes, the maximum relative aerobic power [VO_2_Max (ml/kg/min)] was determined based on the absolute aerobic capacity [VO_2_ (l/min)]; therefore, the VO_2_ (l/min) was acquired specifically for rowers, according to the mathematical models shown below (Klusiewicz et al., 2016):

VO_2_ (l/min) in male sex = {(1,682 + 0.0097) × [Mean Power (Watts) in 2,000-m test performed in Indoor Rowing]}.

VO_2_ (l/min) in female sex = {(1,631 + 0.0088) × [Mean Power (Watts) in 2,000-m test performed in Indoor Rowing]}. (l/min): Liters per minute.

Subsequently, to find the VO_2_Max (ml/kg/min), in both sexes, the VO_2_ (l/min) was converted into millimetres and divided by the athlete’s body weight, according to the following mathematical model (McArdle et al., 2016):

VO_2_ Max (ml/kg/min) = [VO_2_ (l/min) × 1,000]/Body weight (Kg). (ml/kg/min): Millimetres per kilograms per minutes. (Kg): kilograms. VO_2_: Absolute aerobic capacity. (1/min): Litters per minute.

### 2.7 Division of the Athletes’ Training Periods Throughout the Study

**FIGURE 1:**
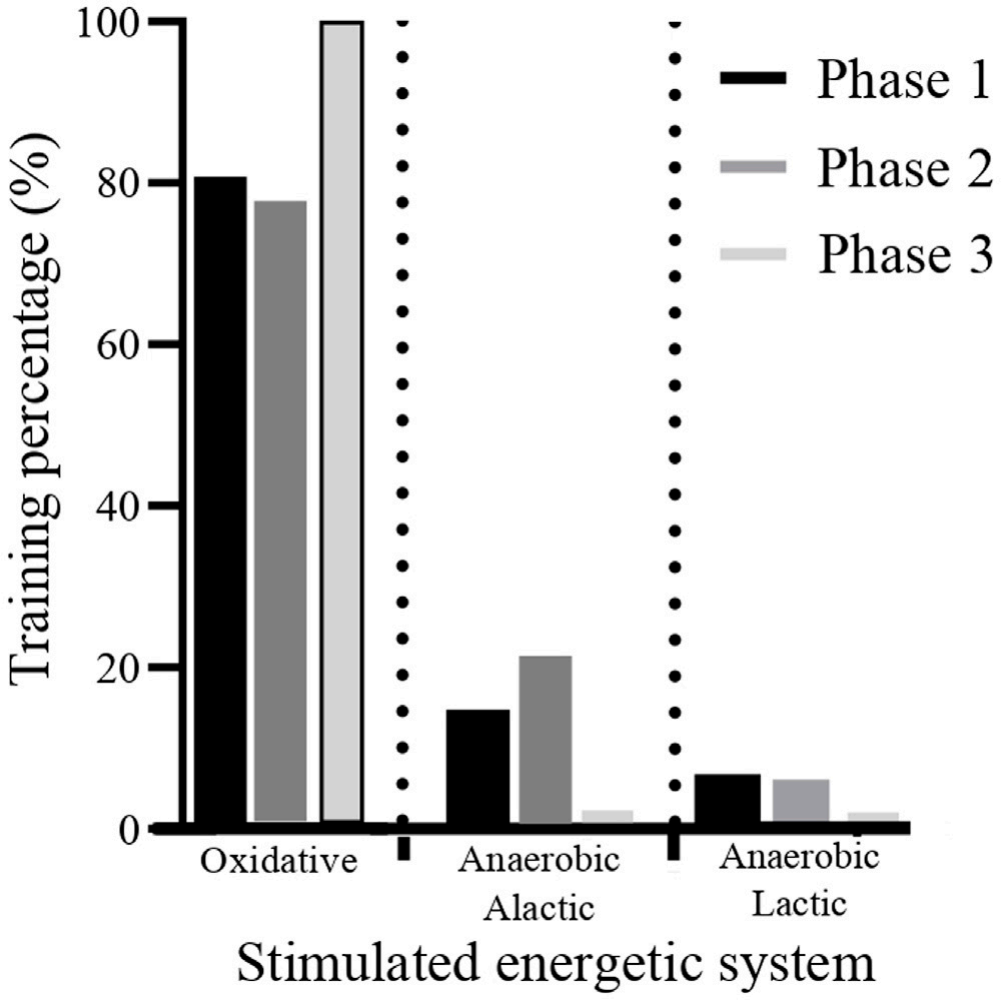
Division of the athletes’ training periods. Phase 1: Pre-competitive period (120–140 km rowing per week). Phase 2: Competitive improvement period (120–140 km rowing per week). Phase 3: Recovery period (90–100 km rowing per week).

Over 3 years of study, the athletes’ training took place based on annual planning based on the main national competition (qualifying regatta for international events) with training volume and intensity based on previous studies which verified the volume and intensity of Rowing training (Seiler and Hetlelid, 2005; Seiler and Kjerland, 2006; Guellich et al., 2009; Bourdon, 2013; Tran et al., 2015). The training was separated into three moments: phase 1 (Pre-competitive period), phase 2 (Competitive improvement period), and phase 3 (Recovery period) (Figure 1). As a result, in the pre-competitive periods, phase 1 (about 8 months before competition), 80% of the athletes’ training was targeted at aerobic stimulus with a focus on increasing hardening activity rowing run <80% pace; heart rate (HR) < 160 b·min^−1^; [Lactate (la) −] <2 mmol·L^−1^ corresponds to work below the ventilatory threshold (VT) 1 (“Zone 1”). The 15% of training for Intensive resistance (lactic) (75%–85% of the rowing running rhythm; FC 156–168 b·min^−1^; [La−] 2–4 mmol·L^−1^) corresponds to the work intensity between VT1 and VT2 (“Zone 2”). The remaining 5% were intended for anaerobic (alactic) stimuli such as 200-m to 500-m shots simulating the start and finish of a competitive race, with highly Intensive and Speed Training stimuli (85%–100% of the pace of running; HR > 180 b·min^−1^; [La−] > 4 mmol·^L−1^) correspond to the work intensity above VT2 (“Zone 3”). The training hours per week were 15 at 18 h per week.

In the competitive improvement period, phase 2 (about 8 weeks), 75% of the athletes’ training was aimed at aerobic stimulus with a focus on a residual permanence of hardening activity (running <80% pace; HR < 160 b·min^−1^; [La−] <2 mmol·L^−1^ corresponds to work below VT1 (“Zone 1”); 20% focused on specific endurance of Intensive Endurance (75%–85% of running pace; 156–168 b·min^−1^; [La−] 2–4 mmol·L^−1^) corresponds to the work intensity between VT1 and VT2 (“Zone 2”), and 5% Highly Intensive stimuli, with a target on the Race Specific Endurance Velocity of 2,000 -m (85%–100% of running pace; HR > 180 b·min^−1^; [La−] > 4 mmol·L^−1^) correspond to the work intensity above VT2 (“Zone 3”). training volume in training hours per week ranged from 14 to 16 total hours of training per week.

In the post-competition moments, phase 3 (8 weeks after the competition), the athletes performed training with a load below VT1 (“Zone 1”), receiving light stimuli with the objective of recovery and technical improvement, with a training volume in hours around 15–16 h a week. Furthermore, the same planning was maintained throughout the 3 years of study.

### 2.8 Statistical Analysis

#### 2.8.1 Data Normality

Data distribution (normal and logarithmic) was tested by Kolmogorov-Smirnov tests, asymmetry, kurtosis Z-score (−1.96 to 1.96), and histogram plotting.

#### 2.8.2 Comparisons

Bonferroni correction was performed before all comparisons. Comparisons between the performance of 2,000-m and VO_2_Max (ml/kg/min) in the different years of the study (2019, 2020, and 2021) were performed using the “two-way Anova” test considering biological maturation factors (pre-PHV, circum-PHV, and post-PHV) and category (U-18 and U-19). Tukey’s post-hoc test identified specific differences. The effect size was verified by the partial squared eta (η2p), considering the magnitude (Cohen, 1992): Small η2*p* ≤ 0.10–0.23; Average η2p from 0.24 to 0.34; Large η2p from 0.35 to 0.44; Very large η2*p* ≥ 0.45. Comparisons of the 1,500-m performance in the different years of the study (2019 and 2020) were performed using the Student-dependent “T” test.

#### 2.8.3 Technical Measurement Error

For the technical error of the intra-examiner anthropometric measurements, the following magnitude was used: acceptable ≤1.0% (Perini et al., 2005). All were performed using the statistical software R (version 4.0.1; R Foundation for Statistical Computing®, Vienna, Austria), and the significance level of *p* < 0.05 was adopted.

## Results

Table 1 shows the sample characterization concerning chronological age, anthropometric variables, distribution of subjects by category, and an absolute number of classifieds for the world championship. It also points out that in males and females, biological maturation was in the circum-PHV classification in the years 2019 and 2020, and post-PHV in the year 2021. We emphasize that for all analyzes, the technical error of measurements was <1.0%.

**TABLE 1:** Sample characterization.

Variables	2019		2020		2021	
	Male	Female	Male	Female	Male	Female
Participants (N)	31	21	31	21	24	21
Chronological Age (Yrs)	16.2 ± 0.5	15.8 ± 0.6	17.2 ± 0.5	16.8 ± 0.6	18.6 ± 0.5	18.2 ± 0.6
Biological maturation (MG-APHV)	-0.1 ± 0.4	0.0 ± 0.2	0.7 ± 0.4	0.3 ± 0.2	1.7 ± 0.3	1.8 ± 0.3
Body weight (Kg)	74.7 ± 6.1	62.0 ± 6.4	74.6 ± 6.2	62.4 ± 6.4	78.7 ± 7.0	63.3 ± 6.0
Stature (cm)	180.5 ± 5.9	169.4 ± 3.1	180.5 ± 6.0	169.4 ± 3.2	182.6 ± 5.53	170.7 ± 2.9
Wingspan (cm)	185.8 ± 7.3	173.0 ± 3.0	185.8 ± 7.4	173.1 ± 3.1	187.6 ± 5.6	173.6 ± 2.9
Category U-17 (%)	45	72	45	72	—	—
Category U-18 (%)	55	28	55	28	42	80
Category U-19 (%)	—	—	—	—	58	20
Classifieds for the world championship (N)	13	15	13	15	10	15
No- Classifieds for the world championship (N)	18	06	18	06	14	06

N, Absolute Number; MG-APHV, Maturational Group−Age for Peak Height Velocity; Kg, kilograms; cm, Centimetres; %, Percentage.

Table 2 reports the comparisons of performance variables at different times in the present study (2019, 2020, and 2021). As a result, there was a significant increase in indoor rowing performance at 2,000-m for males (η2*p* = 0.27, 95% CI: 0.20; 0.30) and females (η2*p* = 0.35, 95% CI: 0.29; 0.36). We observed an increase in competition performance by 2,000-m for males (η2*p* = 0.18, 95% CI: 0.15; 0.25) and females (η2*p* = 0.23, 95% CI: 0.19; 0.26). Furthermore, there was an increase in the mean power (Watts) in Indoor Rowing by 2,000-m for males (η2*p* = 0.11, 95% CI: 0.06; 0.15) and females (η2*p* = 0.21, 95% CI: 0.17; 0.30).Therefore there was an increase in VO_2_Max (ml/kg/min) for males (η2*p* = 0.25, 95% CI: 0.15; 0.30).

**TABLE 2:** Comparisons of rowers’ performance during the study periods.

Variables	Male sample				
	2019	2020	2021	F (2.0)	*p* Value
Indoor rowing performance/2,000-m (min)	7.0 ± 0.4	6.9 ± 0.4	6.4 ± 0.2*	1.73	0.01
Mean power in indoor rowing/2,000-m (watts)	305.3 ± 52.8	306.4 ± 53.0	385.0 ± 27.1*	2.81	0.01
Competition performance/2,000-m (min)	8.0 ± 0.6	7.9 ± 0.6	7.8 ± 0.4*	0.89	0.047
Peak power in competition performance/2,000-m (watts)	360.5 ± 41.1	362.0 ± 42.0	374.1 ± 40.5	0.45	0.09
Mean power in competition performance/2,000-m (watts)	220.6 ± 37.3	219.6 ± 37.0	234.5 ± 19.8	0.05	0.1
Competition performance/1,500-m (min)	6.0 ± 0.1	5.9 ± 0.2	—	—	0.3
Peak power in competition performance/1,500-m (watts)	339.5 ± 26.0	341.0 ± 25.0	—	—	0.1
Mean power in competition performance/1,500-m (watts)	207.1 ± 16.9	210.0 ± 17.0	—	—	0.7
VO2Max (ml/kg/min)	62.0 ± 6.0	62.3 ± 6.4	70.0 ± 5.9*	2.31	<0.001
	Female Sample				
Indoor rowing performance/2,000-m (min)	8.0 ± 0.4	6.9 ± 0.3	6.8 ± 0.2*	8.24	0000.5
Mean power in indoor rowing/2,000-m (watts)	194.0 ± 26.7	311.4 ± 48.3	341.6 ± 88.0*	3.09	0.02
Competition performance/2,000-m (min)	8.6 ± 0.2	8.5 ± 0.2	8.3 ± 0.2*	1.23	0.01
Peak power in competition performance/2,000-m (watts)	277.6 ± 18.0	278.0 ± 18.0	278.0 ± 27.5	0.93	0.06
Mean power in competition performance/2,000-m (watts)	169.6 ± 11.8	170.0 ± 12.0	168.0 ± 10.7	0.75	0.053
Competition performance/1,500-m (min)	6.7 ± 0.2	6.6 ± 0.2	—	—	05
Peak power in competition performance/1,500-m (watts)	260.0 ± 35.0	260.4 ± 35.2	—	—	0.3
Mean power in competition performance/1,500-m (watts)	147.0 ± 13.8	148.0 ± 14.0	—	—	0.1
VO2Max (ml/kg/min)	53.8 ± 4.6	54.0 ± 5.0	54.5 ± 4.0	0.93	0.057

*Statistical difference. VO_2_ Max, Maximum aerobic capacity; ml/kg/min, Millimetres per kilograms per minutes;min, Minutes.

**FIGURE 2:**
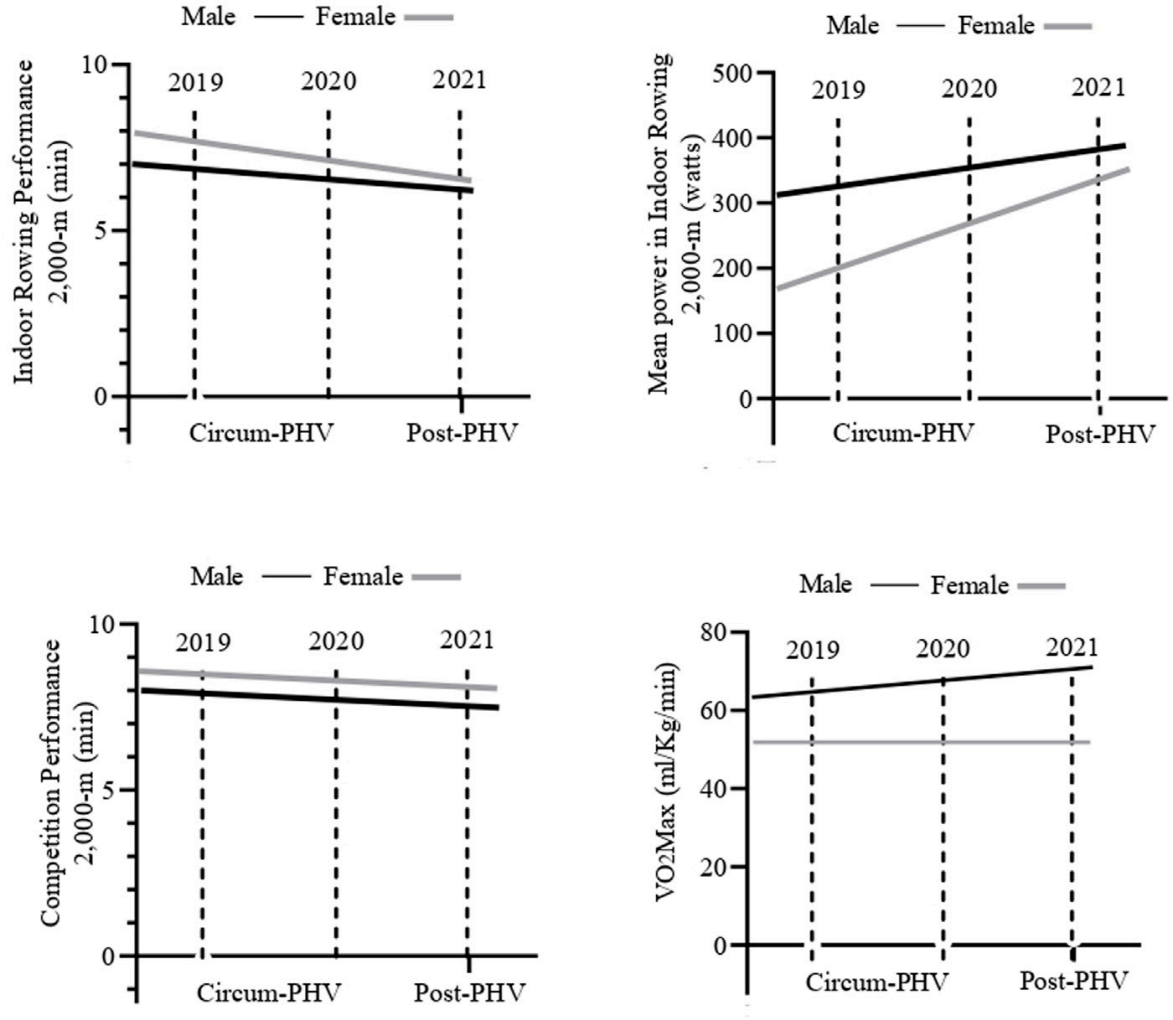
Increased VO_2_ Max (ml/kg/min) performance, indoor rowing, and competition about the advancement of time and biological maturation. VO_2_ Max, Maximum aerobic capacity; ml/kg/min, Millimetres per kilograms per minutes; min, Minutes; PHV, Peak Height Velocity.

Figure 1 shows the behaviour of indoor rowing performance, sports competition performance and VO_2_Max (ml/kg/min) performance in relation to the advancement of time and biological maturation (Figure 2). Regarding the advancement of time (2019–2021), there was an increase in performance in Indoor rowing 2,000-m (Male sex: η2*p* = 0.32, *p* < 0.001; Female sex: η2*p* = 0.36, *p* < 0.001), Mean power (Watts) in indoor rowing 2,000-m (Male sex: η2*p* = 0.27, *p* < 0.001; Female sex: η2*p* = 0.25, *p* < 0.001), Competition performance 2,000-m (Male sex: η2*p* = 0.38, *p* < 0.001; Female sex: η2*p* = 0.34, *p* < 0.001) and VO_2_Max (ml/kg/min) (Male sex: η2*p* = 0.18, *p* = 0.01; Female sex: η2*p* = 0.09, *p* = 0.6).

Regarding the advancement of biological maturation, there was an increase in performance in Indoor rowing 2,000-m (Male sex: η2*p* = 0.33, *p* < 0.001; Female sex: η2*p* = 0.36, *p* < 0.001), Mean Power (Watts) in indoor rowing 2,000-m (Male sex: η2*p* = 0.27, *p* = 0.01; Female sex: η2*p* = 0.23, *p* = 0.01), Competition performance 2,000-m (Male sex: η2*p* = 0.35, *p* < 0.001; Female sex: η2*p* = 0.39, *p* < 0.001). In both sexes, maturation had no significant effect on VO_2_Max (ml/kg/min) (Male sex: η2*p* = 0.009, *p* = 0.1. Female sex: η2*p* = 0.01, *p* = 0.3).

Regarding power (Watts) performance (mean and peak) in 2,000 m competition, and performance in 1,500 m competition (time in seconds and power in watts), no effect of advancing time or advancing biological maturation was found (for both sex). In addition, we verified that in both sexes, the category (U-17, U-18, and U-19) did not exert a significant interaction effect (η2*p* > 0.02, *p* > 0.1).

## Discussion

The present study analysed the influence of biological maturation on sports performance and aerobic and anaerobic power in adolescent rowing athletes. The initial hypothesis was that the advance of biological maturation would not influence aerobic performance but could influence adolescent rowing athletes’ sports and anaerobic performance. Thus, the main findings were: i) The advancement of biological maturation did not affect the increase in VO_2_Max (ml/kg/min) in young elite rowing athletes of both sexes. However, in males, the advance of time had a significant effect on the increase in VO_2_Max (ml/kg/min). ii) The advance of biological maturation had a significant effect on the reduction of the test time and increase of the mean power (Watts) in indoor rowing, and the improvement of performance in sports competition. For this reason, the initial hypothesis of the present study was confirmed.

This research identified that the advancement of biological maturation did not significantly affect VO_2_ Max (ml/kg/min) levels in both sexes. This result can be justified because physiologically, during physical exercise, although the predominance of metabolism in young athletes is oxidative, the glycolytic metabolism becomes more efficient than the aerobic one with the advancement of biological maturation (Rowland, 2008). Subjects in the pre pubertal stage of biological maturation point to an organism with excellent efficiency in producing energy through oxidative pathways, having an efficiency similar to that of hardened adult elite athletes (Birat et al., 2018). During physical exercise, at the beginning of the pubescent stage, the young men show a balance between the efficiency of oxidative and glycolytic metabolism, while in the final stage of the pubescent stage (i.e., transition to post-pubescent), the efficiency of glycolytic metabolism is moderately higher than of the oxidative (Rowland, 2008; McNarry and Barker, 2018). Upon reaching the post pubertal stage, although the predominance of biological metabolism is oxidative, during exercise, the metabolism of young people is characterized by the comprehensive efficiency of the glycolytic energy pathways that are first requested about the oxidative pathways (Rowland, 2008).

Although the advancement of biological maturation did not affect the VO_2_ Max (ml/kg/min), the present study identified that the advancement of time had a significant effect on the VO_2_ Max (ml/kg/min) of male athletes, suggesting that other factors may be associated with the increase in VO_2_ Max (ml/kg/min) in young rowers. In Olympic rowing, during a 2,000-m event, the predominance of the energetic oxidative pathway is 65%–75% and the glycolytic pathway of 25%–35% (Martin & Tomescu, 2017). Thereby, the daily practice of this modality may be enough to promote significant physiological adaptations that increase VO_2_ max (ml/kg/min) levels and physical conditioning in practitioners (Volianitis et al., 2020).

However, although the energy predominance in rowing is oxidative, in the action of rowing, athletes often increase the pace of rows per minute to perform sprints (Gomes et al., 2015), especially at the start of races and in the last meters of the race to cross the finish line (Held et al., 2020), aiming to reduce the running time of the total race. In a previous study, Billat et al. (2009) analysed male rowers aged between 17 and 19 years and concluded that the remaining anaerobic power regulates the running speed in rowers. The authors suggest that the constant changes in speed rhythm during a rowing race can justify the remaining anaerobic power regulating the running speed in rowers aged between 17 and 19 years. However, although these authors have not analysed the stages of biological maturation chronologically, the sample’s age range implies that the subjects were in advanced stages of maturation, where there is greater efficiency of glycolytic metabolism.

The present study found among its main findings that the advance of biological maturation in elite young rowers influenced the increase in anaerobic power and the reduction of the 2,000-m test time in indoor rowing and sports competition. Thus, the anaerobic stimuli performed during a rowing event can significantly contribute to athletes’ overall performance and final classification (Cerasola et al., 2020). Previously, in a study by our group, Almeida-Neto et al. (2020) identified a positive relationship between biological maturation and peak anaerobic power in a 2,000-m run in rowing athletes in the post-PHV maturational stage. In addition, in young athletes of both sexes, biological maturation is identified predictor of power generated by muscle contraction of muscle groups in the lower and upper limbs (Almeida-Neto et al., 2021). Olympic rowing is a sport that constantly uses the muscular power of the upper limbs to overcome the resistance of the aquatic environment and thus move the vessel (Gee et al., 2016). In addition, local blood flow (of active musculature) is identified as a determinant of the overall efficiency of the oxygen transport system in rowers (Secher, 1983). These characteristics suggest that we must consider several factors that may interact with rowers’ performance.

Previously, Dantas et al. (2018) found significant relationships between the advancement of biological maturation and the increase in power of upper and lower limbs in rowers of both sexes. However, through an allometric model, Diry et al. (2020) verified that concerning the factors that influence the glycolytic metabolism in adolescents, the total lean mass seems to exert a more significant effect than biological maturation. Further, it was found that in young elite rowers of both sexes in the post-PHV stage of biological maturation, muscle power is superior to that of other athletes in late maturation stages (i.e., pre pubertal and pubescent) (Almeida-Neto et al., 2020b).

Nonetheless, we need to emphasize that the advancement of biological maturation will promote the increase of anabolic androgenic hormones (i.e., testosterone, estradiol, progesterone, and dehydroepiandrosterone), which are closely linked with the increase in lean body mass (La Perle & Dintzis, 2018; Sperling, 2020). It was previously verified by Durkalec-Michalski et al. (2019) that lean mass is closely related to the level of aerobic and anaerobic capacity of rowers. Likewise, it was pointed out that young elite athletes of both sexes (Almeida-Neto et al., 2020b) and non-elite males (Almeida-Neto et al., 2020c) with biological maturation in advanced stages point to greater lean body mass levels compared to their delayed maturation peers. Consequently, pointing to higher androgenic hormone levels and better efficiency of glycolytic metabolism, thus generating higher levels of muscle power (Rowland, 2008; Almeida-Neto et al., 2020c).

Based on this discussion, we emphasize that the present study brings a strong point that it gathers information about the performance of elite rowers concerning the circum-PHV and post-PHV biological maturation stages, exposing the performance behaviour in the transition between referred to maturational stages. However, our main limitation was that at the beginning of the study, the participants were in the initial stage of the circum-PHV stage, which made it impossible for us to analyse the performance behaviour during the transition from the pre-PHV to the circum-PHV stage. In addition, VO_2_ Max was estimated and not obtained directly (gas analysis), results by gas analyzers may differ from those pointed out by the present study.

Therefore, the present study contributes to sports training, emphasizing that biological maturation must be considered when carrying out longitudinal training planning in adolescent rowing athletes of both sexes. In practical terms, considering that rowing will naturally stimulate the increase in VO_2_Max (ml/kg/min) levels and that anaerobic power (Watts) is related to competitive performance, in the circum-PHV stage, the anaerobic stimuli during training can be increased to enhance the efficiency of this mechanism. While in the post-PHV stage, the anaerobic stimuli during training can be reduced due to the efficiency of the glycolytic pathways being more efficient than before. For these reasons, we suggest that for circum-PHV athletes, training can focus on maintaining endurance capacity and increasing anaerobic power. As for post-PHV athletes, we suggest maintaining anaerobic power (Watts) and focusing on increasing the capacity to endurance.

## Conclusion

Our study concluded that the advance of biological maturation influences the anaerobic performance of young rowing athletes of both genders, contributing to the reduction of the execution time and increase of the mean anaerobic power (watts) during a performance in a 2,000-m test (performed in indoor rowing). As well as, for reduction of the execution time in 2,000-m event held in a single scull boat during sports competition. It is also concluded that the advance of biological maturation did not influence gain in aerobic power (VO_2_Max (ml/kg/min)) in adolescent rowing athletes of both genders.

## Data Availability

The datasets presented in this study can be found in online repositories. The names of the repository/repositories and accession number(s) can be found below: The data used in this study are publicly available on the platform https://figshare.com/ under DOI 10.6084/m9.figshare.16373142.
